# Prognostic significance of KRAS G12C versus non-G12C RAS mutations in metastatic colorectal cancer: a systematic review and meta-analysis

**DOI:** 10.1093/oncolo/oyag219

**Published:** 2026-06-02

**Authors:** Mohamed M Khamis, Mahsa Shirani Lapari, Omar Alkharabsheh, Maun R Baral, Ibrahim Halil Sahin, Anita Archwamety, Belal Firwana, Girijesh Kumar Patel, Ajay Singh, Sameer Al Diffalha, Upender Manne, Moh’d Khushman

**Affiliations:** Internal Medicine, Mercy Hospital, St. Louis, MO, 63141, United States; Division of Endocrinology, Department of Medicine, Johns Hopkins University School of Medicine, Baltimore, MD, 21205, United States; Hematology-Oncology, University of Cincinnati, Cincinnati, OH, 45221, United States; Medical Oncology, Washington University in St. Louis, St. Louis, MO, 63110, United States; Division of Hematology and Oncology, Department of Internal Medicine, University of Michigan Medical School, Rogel Cancer Center, Ann Arbor, MI, 48109, United States; Medical Oncology, Siriraj Hospital, Bangkok, 10700, Thailand; Medical Oncology, Heartland Cancer Research, Missouri Baptist Medical Center, Saint Louis, MO, 63131, United States; Department of Biotechnology, Motilal Nehru National Institute of Technology, Prayagraj, UP, 211004, India; Cell and Molecular Biology, University of Mississippi Medical Center, Jackson, MS, 39216, United States; Pathology, The University of Alabama at Birmingham, O’Neal comprehensive Cancer Center, Birmingham, AL, 35294, United States; Pathology, The University of Alabama at Birmingham, O’Neal comprehensive Cancer Center, Birmingham, AL, 35294, United States; Medical Oncology, Washington University in St. Louis, St. Louis, MO, 63110, United States; Siteman Cancer Center, St. Louis, MO, 63110, United States

**Keywords:** colorectal cancer, KRAS G12C mutation, prognosis, overall survival, meta-analysis

## Abstract

**Background:**

KRAS G12C mutations occur in approximately 3%-4% of metastatic colorectal cancer (mCRC) cases. While the introduction of KRAS G12C inhibitors has transformed the therapeutic landscape for this molecular subset, conflicting evidence exists regarding the independent prognostic impact of this mutation in inhibitor-naïve settings. Small sample sizes and methodological heterogeneity have limited individual studies, precluding definitive conclusions. To address this knowledge gap, we conducted a systematic review and meta-analysis to establish the prognostic significance of KRAS G12C mutations in mCRC.

**Methods:**

A comprehensive systematic literature search was conducted across PubMed, Google Scholar, and Cochrane Library through August 2025. Studies comparing overall survival between KRAS G12C and non-G12C RAS-mutant mCRC were included. Hazard ratios (HRs) were extracted or calculated from reconstructed individual patient data. Pooled analyses employed random-effects models. Quality assessment utilized the Newcastle–Ottawa Scale.

**Results:**

Fourteen retrospective studies encompassing 9308 patients (903 KRAS G12C, 8405 non-G12C RAS) were included. The pooled analysis demonstrated that KRAS G12C mutations confer significantly worse prognosis, with a 28% increased risk of mortality compared to non-G12C RAS mutations (HR = 1.28, 95% CI, 1.10-1.48; *p* = .0015). A meta-analysis of difference in medians showed a pooled median overall survival (mOS) difference of −4.5 months (95% CI, −9.1 to 0.0; *P* = .05) and a pooled median progression-free survival (mPFS) difference of −1.3 months (95% CI, −2.4 to −0.1; *P* = .03) for KRAS G12C versus non-G12C patients. Moderate heterogeneity was observed (*I*^2^ = 54.3%). Sensitivity analysis restricted to high-quality studies confirmed these findings (HR = 1.31, 95% CI, 1.11-1.54). No publication bias was detected.

**Conclusions:**

KRAS G12C mutations represent an independent adverse prognostic biomarker in mCRC, with a statistically significant 28% increased risk of mortality compared to other RAS mutations. The consistent HR across multiple sensitivity analyses supports a true prognostic effect. These findings have important implications for patient counseling and risk stratification. While the poor prognosis may provide rationale for prioritizing trial enrollment, translation into therapeutic decision-making requires caution, as prospective data demonstrating benefit from earlier use of KRAS G12C inhibitors are lacking.

Implications for Practice This meta-analysis establishes KRAS G12C as an independent adverse prognostic biomarker in metastatic colorectal cancer, conferring a 28% higher mortality risk compared to other RAS mutations (hazard ratio [HR] = 1.28, 95% CI, 1.10-1.48). A meta-analysis of median survival differences demonstrated a significant 1.3-month shorter median progression-free survival for KRAS G12C patients (*P* = .03), with a trend toward 4.5 months shorter median overall survival (*P* = .05), providing clinically interpretable estimates that complement the pooled HR. These findings inform risk-stratified patient counseling and may support prioritization of this high-risk population for clinical trial enrollment. However, the prognostic significance under standard chemotherapy does not automatically translate to predictive significance for targeted therapies. The potential benefits of earlier integration of KRAS G12C inhibitors must be weighed against risks including toxicity and potential compromise of standard therapy delivery. Prospective randomized trials are needed to determine optimal treatment sequencing for this molecular subset.

## Introduction

Metastatic colorectal cancer (mCRC) remains a formidable global health challenge, accounting for more than 1.85 million new cases and 850 000 deaths annually worldwide.^1^ Approximately 20% of patients present with metastatic disease at diagnosis, and an additional 25% of those initially diagnosed with localized disease will eventually develop metastases.^1^ Despite significant advances in systemic therapy over the past two decades, the prognosis for patients with unresectable mCRC remains poor, with fewer than 20% surviving beyond 5 years from diagnosis.^1^ The molecular landscape of mCRC is characterized by a complex array of genetic alterations, with activating mutations in the RAS family of oncogenes—particularly KRAS and NRAS—representing one of the most prevalent oncogenic drivers, collectively present in 35%-50% of cases.^1^^,^^2^ These mutations are central to colorectal tumorigenesis, driving constitutive activation of the MAPK and PI3K signaling pathways, and have been established as robust predictors of resistance to anti-EGFR monoclonal antibodies such as cetuximab and panitumumab.^1^^,^^2^ Consequently, the American Society of Clinical Oncology and other major international societies recommend universal RAS testing in mCRC to guide therapy selection, as only patients with RAS wild-type tumors derive clinical benefit from anti-EGFR agents.^1^^,^^2^

Within this molecular framework, the KRAS G12C mutation—a glycine-to-cysteine substitution at codon 12—has emerged as a clinically significant and actionable molecular subset, present in approximately 3%-4% of mCRC cases.^3^ The development of covalent KRAS G12C inhibitors, including sotorasib and adagrasib, has fundamentally transformed the therapeutic landscape for this population, offering the first targeted treatment options for a group previously limited to cytotoxic chemotherapy and anti-angiogenic agents.^4–6^ Recent phase III clinical trial data have established the combination of KRAS G12C inhibitors with anti-EGFR monoclonal antibodies as a new standard of care in patients with mCRC, demonstrating superior progression-free survival (PFS) compared to standard late-line therapies.^4^^,^^5^ These developments represent a paradigm shift in the management of KRAS G12C-mutant mCRC, transitioning from an “undruggable” target to a therapeutically actionable molecular subset with defined treatment algorithms.^6^

However, while the therapeutic role of KRAS G12C inhibition is now well-established in refractory disease, the independent prognostic significance of this mutation in inhibitor-naïve treatment settings remains a subject of considerable scientific debate and clinical uncertainty.^3^^,^^7^^,^^8^ Several individual studies have suggested that KRAS G12C confers a worse prognosis compared to other RAS mutations, with quantitative evidence demonstrating shorter overall survival (ranging from 16.1 to 21.1 months versus 18.3 to 27.3 months for non-G12C mutations) and PFS in various clinical contexts.^7^^,^^9^ Notably, the multicenter retrospective study by Chida et al demonstrated that KRAS G12C was independently associated with shorter overall survival (HR = 1.42, 95% CI, 1.01-2.00, *P* = .044) after adjustment for clinical and molecular confounders.^7^ Similarly, pooled analyses from randomized trials, including the AIO Colorectal Cancer Study Group analysis, have identified KRAS G12C as potentially the worst prognostic variant among KRAS mutations.^10^ The STORIA analysis further corroborated these findings, identifying KRAS G12C as being associated with particularly poor outcomes.^11^

Despite this emerging evidence, the literature remains characterized by inconsistent results, small sample sizes reflecting the low prevalence of KRAS G12C (3%-4% of mCRC cases), and significant methodological limitations.^3^^,^^7^^,^^8^ Most available evidence derives from retrospective observational studies with limited statistical power for the G12C subgroup and incomplete adjustment for potential clinical and molecular confounding factors.^7^^,^^8^ The heterogeneity in study populations, treatment regimens, outcome definitions, and analytical approaches has precluded definitive conclusions about the independent prognostic impact of KRAS G12C mutation.^3^^,^^8^ Furthermore, systematic reviews to date have been primarily descriptive in nature, lacking the quantitative synthesis necessary to resolve these conflicting findings and establish evidence-based clinical recommendations.^3^

This critical knowledge gap has important clinical implications, as accurate prognostic stratification is essential for treatment selection, patient counseling, and clinical trial design in the era of precision oncology. The identification of KRAS G12C as a high-risk molecular subset would support more intensive monitoring strategies, earlier consideration of clinical trial enrollment, and prioritization of access to targeted therapies. Conversely, if KRAS G12C does not confer independent prognostic significance, treatment algorithms and risk stratification models would need to focus on other molecular and clinical determinants of outcome.

To address this critical gap in knowledge, we conducted a comprehensive systematic review and meta-analysis to definitively establish the prognostic significance of KRAS G12C mutation in mCRC.

## Methods

### Search strategy and study selection

A comprehensive systematic literature search was conducted across multiple electronic databases including PubMed, Google Scholar, and the Cochrane Library from inception through August 2025. Detailed search strategies are provided in Appendix S1 in Supplementary Material. The search excluded reviews, editorials, comments, and letters. Only studies published in English were included. Two independent reviewers screened titles and abstracts, with full-text articles retrieved for potentially eligible studies. Disagreements were resolved through discussion and, when necessary, consultation with a third reviewer. This systematic review was conducted in accordance with the Preferred Reporting Items for Systematic Reviews and Meta-Analyses (PRISMA) guidelines.

### Inclusion and exclusion criteria

Studies were included in the meta-analysis if they met all of the following criteria: (1) population: patients with mCRC, also described as “advanced” or “Stage IV” disease; (2) mutation status: studies must clearly identify and report on a cohort of patients with KRAS G12C mutations and provide comparative data for patients with non-G12C RAS mutations (including other KRAS or NRAS mutations), with mutation groups reported separately rather than aggregated; (3) outcomes: studies must report overall survival (OS) data in at least one of the following formats: HRs with 95% CI (preferred), Kaplan–Meier (KM) survival curves of sufficient quality for HR estimation (acceptable), or median survival times with patient numbers (minimum acceptable).

Studies were excluded if they (1) focused on non-mCRC or combined multiple cancer types without distinct mCRC subgroup data; (2) included patients treated with KRAS G12C-specific inhibitors (eg, sotorasib, adagrasib) unless survival data for inhibitor-naïve subgroups were separately reported; (3) failed to specifically report KRAS G12C mutation data or lacked appropriate comparison groups; (4) were case reports, small case series (<10 patients in G12C arm), commentaries, narrative reviews, editorials, or conference abstracts without sufficient extractable data.

### Data extraction

A standardized data collection form was used to extract relevant information from each of the included studies. Two independent reviewers performed the data extraction. The completed forms were then cross-verified to ensure accuracy, and any discrepancies were resolved through discussion and consensus.

### Risk of bias assessment

The methodological quality and risk of bias for each included study were independently assessed by two reviewers using the Newcastle-Ottawa Scale (NOS) for non-randomized cohort studies. This scale evaluates study quality across three key domains: the selection of study groups (up to 4 points), the comparability of the groups (up to 2 points), and the ascertainment of the outcome of interest (up to 3 points). Studies were scored on a scale from 0 to 9. Based on the total score, each study was categorized as representing either “high quality” (score of 7-9), “moderate quality” (score of 5-6), or “low quality” (score of 0-4) study, using established cutoffs from previous meta-analyses. This categorization was used in subsequent sensitivity analyses to assess the robustness of our findings.

### Statistical analysis

The primary effect measure for this meta-analysis was the HR for overall survival, comparing the prognosis of patients with KRAS G12C mutations to those with non-G12C RAS mutations. For studies that directly reported an HR and its 95% CI the natural logarithm of the HR (logHR) and its corresponding standard error (SE) were calculated for pooling. In cases where an HR was not provided, KM survival curves were digitized to reconstruct individual patient data (IPD) using the algorithm described by Guyot et al. A Cox proportional hazards model was then fitted to the reconstructed IPD to estimate the study-specific logHR and SE. To provide clinically interpretable median survival estimates, we additionally performed a random-effects meta-analysis of the difference in median OS and median PFS between KRAS G12C and non-G12C groups using the generic inverse-variance method. For each study reporting median survival for both groups, the difference in medians (G12C minus non-G12C) was calculated.

Pooled analyses were conducted using a random-effects model (DerSimonian–Laird method) to account for between-study heterogeneity. Statistical heterogeneity was quantified using Cochran’s Q statistic, the *I*^2^ statistic (describing percentage of variability due to heterogeneity rather than chance), and τ^2^ (between-study variance). *I*^2^ values of 25%, 50%, and 75% were considered to represent low, moderate, and high heterogeneity, respectively. Forest plots were generated to display study-specific and pooled HRs with 95% CIs. Statistical significance was set at α = 0.05 for all analyses.

### Sensitivity and publication bias analysis

To assess the robustness of the pooled estimate, a sensitivity analysis was performed by repeating the meta-analysis exclusively on studies deemed to be of high quality (i.e. those with a NOS score of 7 or higher). The potential for publication bias was investigated through visual inspection of a funnel plot for asymmetry. This was further assessed statistically using both Begg’s rank correlation test and Egger’s linear regression test, with a *P* value <.05 considered suggestive of significant publication bias. A leave-one-out analysis was performed, sequentially omitting each study to assess whether any single study disproportionately influenced the pooled effect estimate. All statistical analyses were performed using R version 4.3.1 (R Foundation for Statistical Computing, Vienna, Austria). The meta-analysis was conducted primarily using the meta package, with data manipulation and visualization supported by the tidyverse and ggplot2 packages. The IPD from KM and survival packages were used for the reconstruction of IPD from KM curves and subsequent survival analysis.

## Results

### Study selection and characteristics

**Figure 1.**
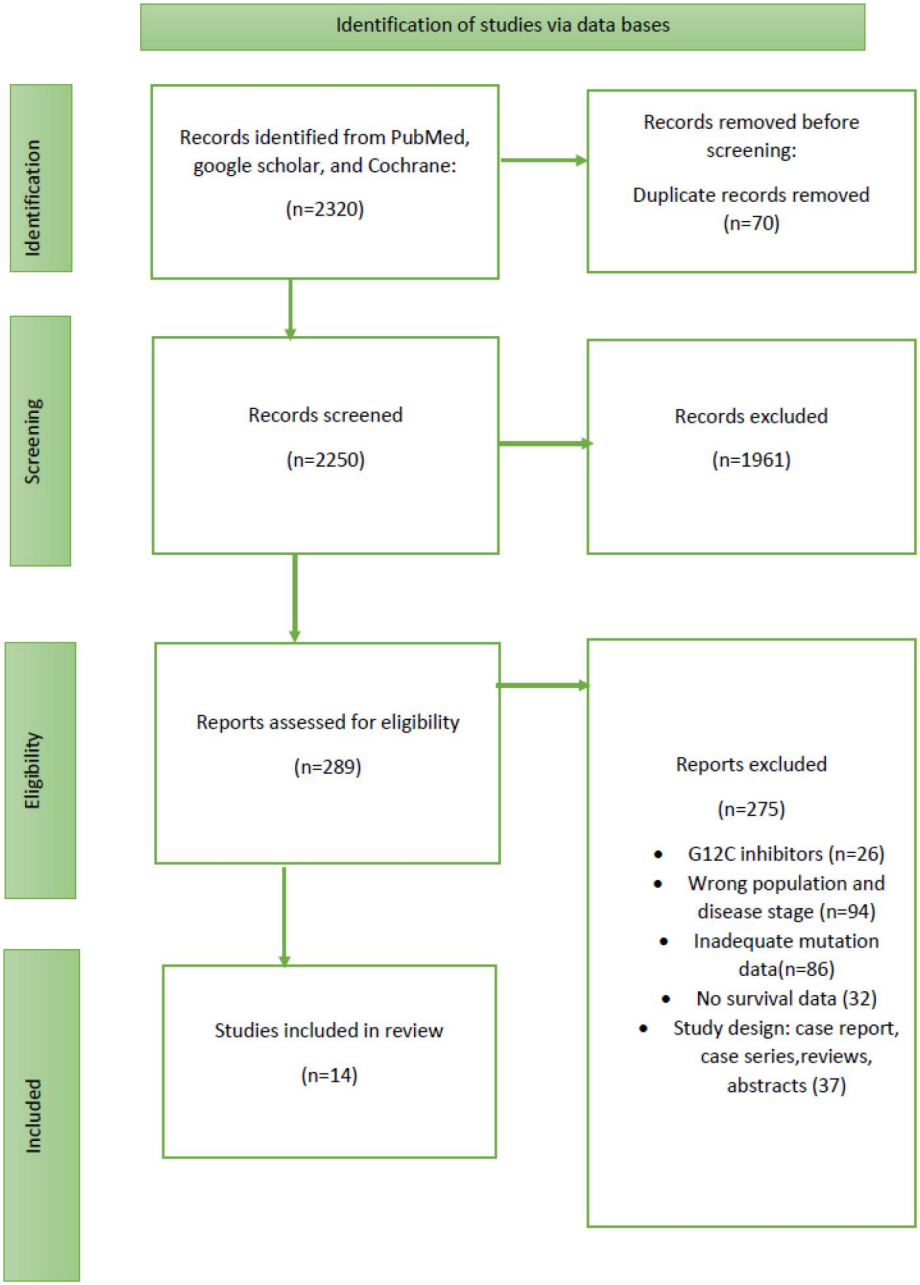
PRISMA flow diagram for study selection.

The study selection process is detailed in the PRISMA flow diagram (Figure 1). The initial literature search yielded 289 records, from which 14 retrospective studies were ultimately included. These studies collectively enrolled 9308 patients with mCRC, comprising 903 patients with a KRAS G12C mutation and 8 405 with non-G12C RAS mutations. HRs for overall survival were directly extracted from eight studies and calculated from reconstructed IPD for the remaining six studies. The detailed characteristics of each study are presented in Table 1.

**Table 1.** Baseline characteristics and risk of bias assessment of studies included in the meta-analysis.

Study	Total patients (*N*)	KRAS G12C (*n*)	Non-G12C RAS (*n*)	Median age (G12C cohort)	Chemotherapy regimen	Line of therapy	% Female (G12C cohort)	HR source	NOS score
**Chida et al.^7^**	696	45	651	65	FOLFOX/CAPOX/FOLFIRI/FOLFOXIRI	First	53.3	Reported HR	9
**Contreras-Toledo et al.^12^**	492	33	459	NR	FOLFIRI, Irinotecan-based	Second	NR	IPD Recon.	9
**Fakih et al.^9^**	3185	238	2947	59	Oxaliplatin + FP (60%), Irinotecan + FP (10%)	First	43.3	IPD Recon.	9
**Giampieri et al.^8^**	120	15	105	NR	FOLFIRI/FOLFOX/XELOX	First	67	Reported HR	9
**Henry et al.^13^**	907	187	720	56	Oxaliplatin- or Irinotecan-based	First	51.9	IPD Recon.	8
**Lutz et al.^14^**	321	24	297	NR	Mixed regimens	Mixed	NR	IPD Recon.	6
**Koulouridi et al.^15^**	567	28	539	64	Irinotecan-based (53%), LOHP-based (43%)	First	32.1	IPD Recon.	6
**Lavacchi et al.^16^**	182	13	169	NR	Standard first-line chemotherapy	First	NR	IPD Recon.	7
**Li et al.^17^**	643	30	613	62.5	Irinotecan-based (63%), Capecitabine (20%)	First	40	Reported HR	8
**Osterlund et al.^18^**	984	103	881	67	5-FU, Oxaliplatin, Irinotecan	Mixed	45	Reported HR	9
**Ottaiano et al.^11^**	157	13	144	NR	FOLFOX/CAPOX, FOLFIRI	First+	61.5	Reported HR	9
**Peeters et al.^19^**	126	12	114	NR	FOLFOX4, FOLFIRI	First/Second/Third	NR	Reported HR	7
**Schirripa et al.^20^**	839	145	694	64	NR	NR	29	Reported HR	8
**Usón et al.^21^**	89	17	72	NR	5-FU, Oxaliplatin, Irinotecan	Mixed	NR	Reported HR	7
**Total**	9 308	903	8 405						

All studies are retrospective. HR source indicates whether HRs were directly extracted from published data or calculated from reconstructed individual patient data using the Kaplan–Meier curves. NOS scores range from 0-9, with ≥7 considered high quality.

Abbreviations: FP, fluoropyrimidine; IPD Recon, individual patient data reconstruction; LOHP, oxaliplatin; NOS, Newcastle–Ottawa Scale; NR, not reported;.

### Meta-analysis of overall survival

**Figure 2.**
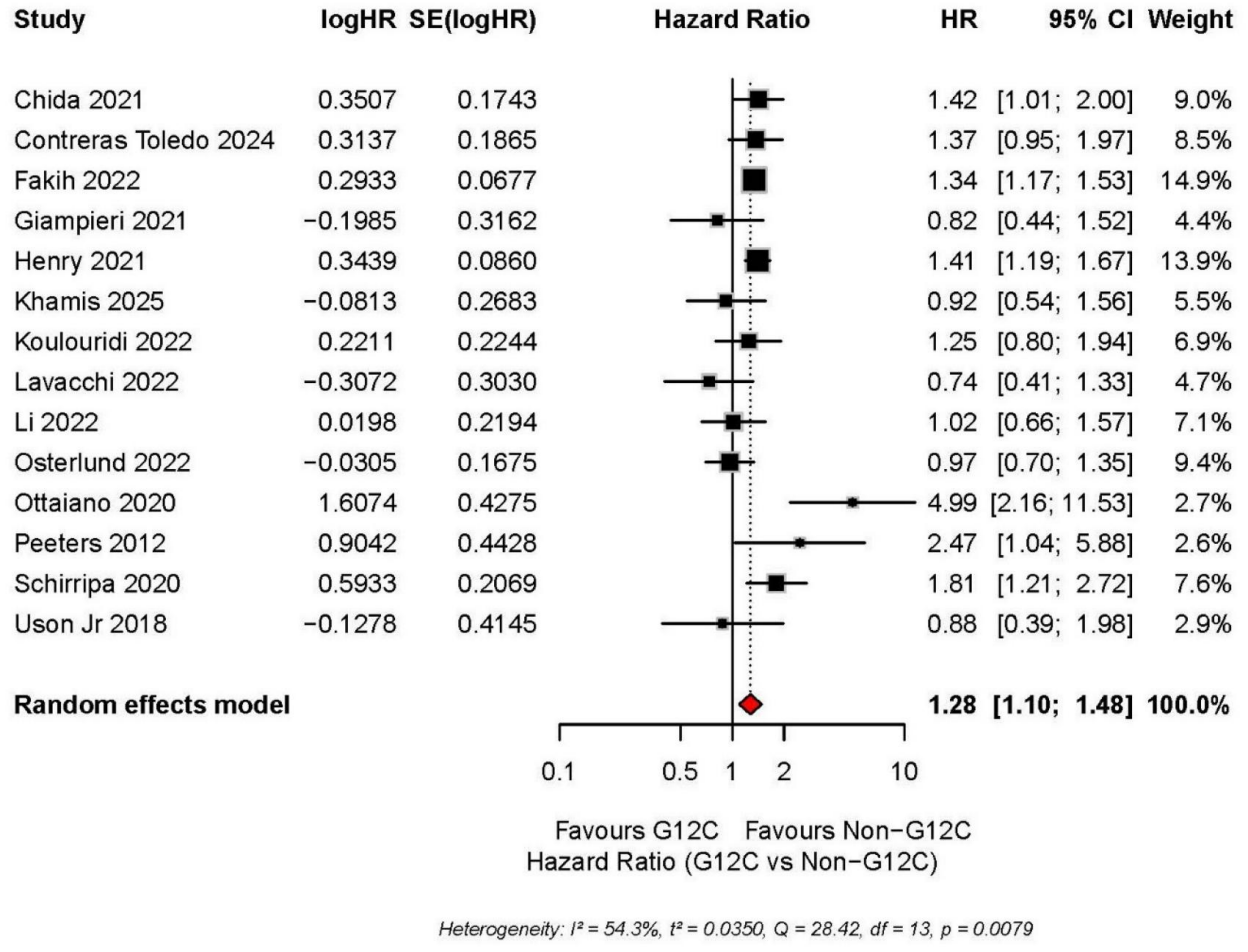
Forest plot of overall survival—KRAS G12C vs non-G12C RAS mutations.

The primary meta-analysis demonstrated that the KRAS G12C mutation is associated with a significantly worse prognosis compared to non-G12C RAS mutations. The pooled HR was 1.28 (95% CI, 1.10–1.48; *P* = .0015), indicating a 28% increased risk of mortality for patients with the G12C mutation (Figure 2). Moderate heterogeneity was observed among the studies (*I*^2^ = 54.3%; *P* = .008), suggesting some variability in effect sizes across studies that may be attributed to differences in study populations, treatment regimens, or follow-up periods. Individual study HRs varied from 0.74 to 4.99, with most studies showing a trend toward shorter survival for the G12C cohorts. Notably, large retrospective cohorts such as Fakih et al.^9^ and Henry et al.^13^ contributed the greatest statistical weight and showed unfavorable outcomes for KRAS G12C.

### Median survival analysis

**Figure 3.**
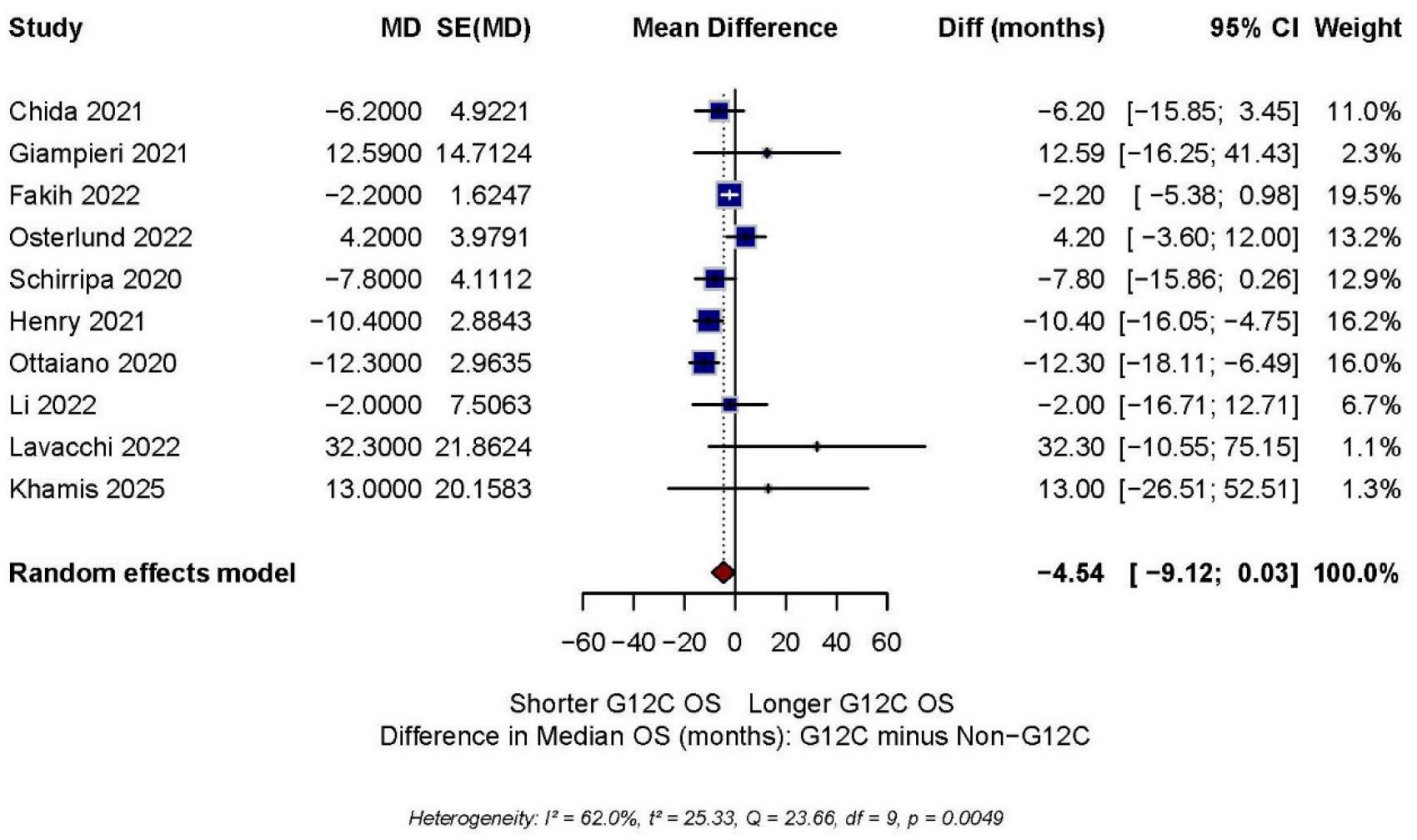
Forest plot of difference in median overall survival between KRAS G12C and non-G12C mCRC patients.

To provide clinically interpretable median survival estimates, we performed a random-effects meta-analysis of the difference in medians between KRAS G12C and non-G12C groups. Among 10 studies reporting median OS for both groups, the pooled difference in median OS was −4.5 months (95% CI, −9.1 to 0.0; *P* = .0515), indicating a trend toward shorter median OS for KRAS G12C patients, with moderate heterogeneity (*I*^2^ = 62.0%) (Figure 3). Among five studies reporting median PFS for both groups, the pooled difference in median PFS was −1.3 months (95% CI, −2.4 to −0.1; *P* = .0319), representing a statistically significant shorter median PFS for KRAS G12C patients, with no heterogeneity (*I*^2^ = 0%). Individual study median survival data are presented in Table S2. The wide variability in individual study medians—with KRAS G12C mOS ranging from 5.0 to 65.0 months and non-G12C mOS from 17.3 to 52.0 months—reflects the clinical heterogeneity of the included populations. While the pooled mOS difference approached but did not reach statistical significance, the significant mPFS difference and the significant pooled HR = 1.28 together provide convergent evidence of worse outcomes for KRAS G12C patients. The pooled HR, which accounts for the full survival distribution and censoring patterns, remains the primary outcome measure for this analysis.

### Quality assessment

The methodological quality of the included studies was generally high, with the NOS scores ranging from 6 to 9 (mean score: 7.9). Twelve of the fourteen studies (86%) were rated as “high quality” (NOS score ≥ 7), and two were rated as “moderate quality” (NOS score of 6). No studies were categorized as “low quality.” A detailed item-by-item breakdown of the NOS scores is provided in Table S1, and a visual summary of the assessment is presented in Figure S1.

### Sensitivity and publication bias analyses

**Figure 4.**
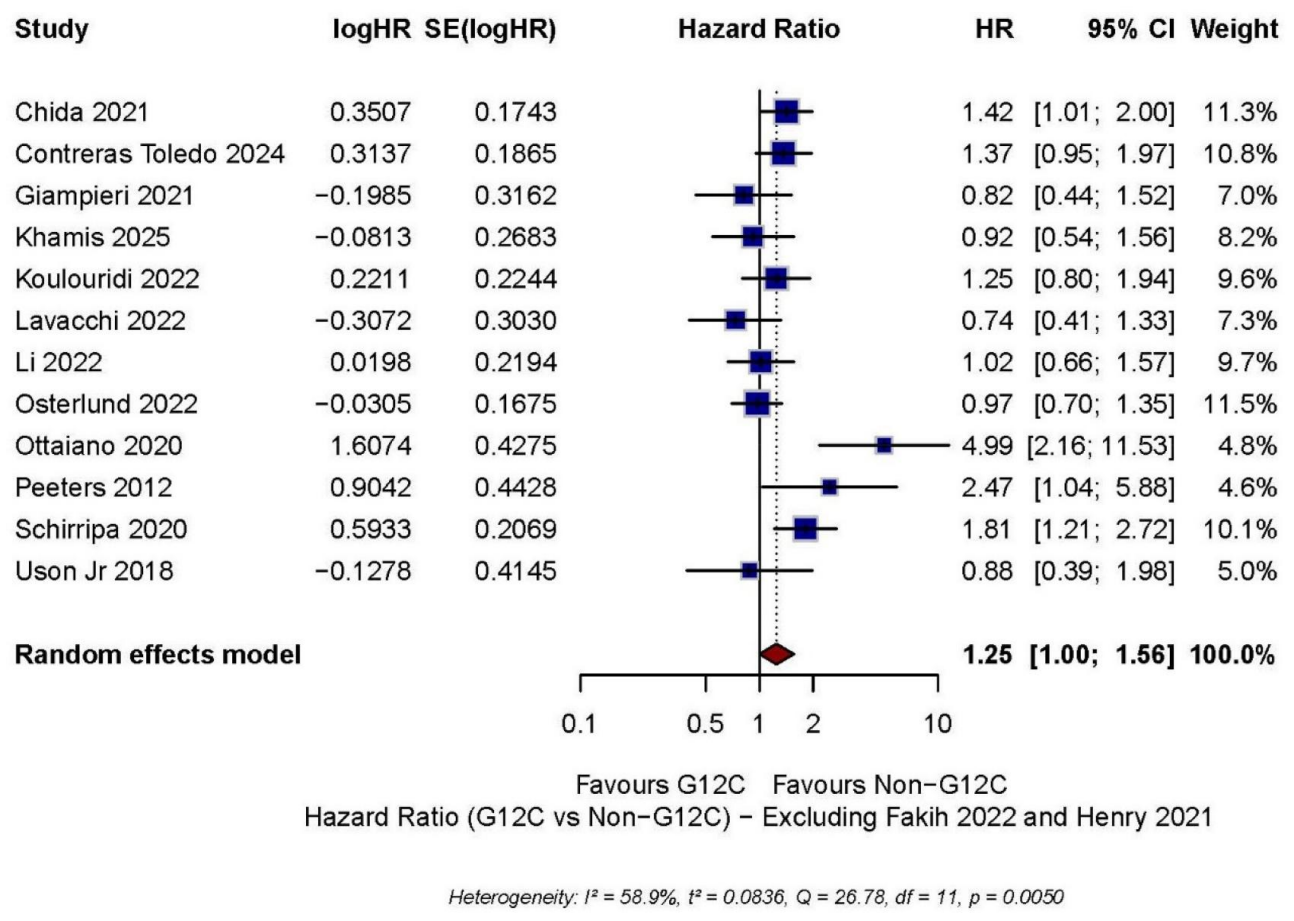
Sensitivity analysis: hazard ratio for overall survival excluding Fakih et al.^9^ and Henry et al.^13^

The primary finding was robust across multiple sensitivity analyses. First, restricting analysis to the 12 high-quality studies, (NOS ≥ 7) yielded a consistent and significant pooled HR of 1.31 (95% CI, 1.11-1.54) (Figure S2). Second, given that the two largest studies (Fakih et al.^9^, *n* = 3,185 and Henry et al.^13^, *n* = 907) contributed approximately 44% of the total patient population and both demonstrated unfavorable outcomes for KRAS G12C, we performed an additional sensitivity analysis excluding these studies. This analysis of the remaining 12 studies confirmed the robustness of our findings, with a pooled HR of 1.25 (95% CI, 1.00-1.56; *P* = .0493) (Figure 4), demonstrating that the observed prognostic effect is not solely driven by these large cohorts. A leave-one-out analysis also demonstrated the stability of the pooled estimate, with no single study exerting undue influence on the overall result. Furthermore, no evidence of significant publication bias was detected. The funnel plot was visually symmetrical (Figure S3), which was supported by non-significant results from both Begg’s test (*P* = 0.78) and Egger’s test (*P* = 0.74). The results of all analyses are summarized in Table 2.

**Table 2.** Summary of primary and sensitivity meta-analysis results for overall survival.

Analysis group	No. of studies	Pooled HR	95% CI	*P* value	Heterogeneity (*I²*)	*P* value for heterogeneity
**All studies**	14	1.28	1.10–1.48	.0015	54.30%	.008
**High-quality studies (NOS ≥ 7)**	12	1.31	1.11–1.54	.0016	58.60%	.005
**Excluding Fakih et al.^9^ and Henry et al.^13^**	12	1.25	1.00–1.56	.0493	58.9%	.005

Abbreviations: CI, confidence interval; HR, hazard ratio; NOS, Newcastle–Ottawa Scale.

## Discussion

It is already well-established that *RAS* mutations confer poor overall prognosis and resistance to anti-EGFR therapy in patients with mCRC.^1^^,^^2^ On a more granular level, several studies suggested that *KRAS G12C* mutations confer worse prognosis compared to RAS non-G12C mutations. Such observation was not reported in all studies and hence there is currently a debate and uncertainty about the real prognostic significance of *KRAS G12C* in mCRC.

The present meta-analysis provides the first quantitative synthesis and analysis of this kind to help with resolving the debate and uncertainty regarding the prognostic significance of KRAS G12C compared to RAS non-G12C. One clinical implication of this meta-analysis is that it highlights KRAS G12C mutation as a prognostic biomarker in mCRC. Our pooled analysis of 14 retrospective studies encompassing 9,308 patients demonstrates a statistically significant 28% increased risk of mortality (HR = 1.28, 95% CI, 1.10-1.48; *P* = .0015) for patients harboring *KRAS G12C* mutations compared to those with *non-G12C KRAS* mutations. This finding directly addresses the inconsistent results reported in individual studies, where sample sizes were insufficient to detect meaningful differences due to the low prevalence of KRAS G12C (3%-4% of mCRC cases), and provides robust evidence that transcends the methodological limitations of single-center analyses.^3^^,^^7^^,^^8^

KRAS G12C mutations in colorectal cancer are associated with worse prognosis through several distinct biological mechanisms. While the glycine-to-cysteine substitution at codon 12 impairs GTP hydrolysis and shifts KRAS toward the active GTP-bound state to drive oncogenic signaling, KRAS G12C paradoxically retains greater intrinsic GTP hydrolysis activity compared to other codon 12 mutations like G12V or G12R.^5^^,^^22^ This creates a unique vulnerability that enables covalent inhibitor binding but also renders KRAS G12C-mutant colorectal cancers highly susceptible to rapid compensatory signaling through EGFR pathway reactivation, which drives aggressive tumor biology and therapeutic resistance.^4^^,^^6^ Unlike non-small cell lung cancer, KRAS G12C colorectal cancer models exhibit high basal receptor tyrosine kinase activation and are highly responsive to growth factor stimulation, leading to rapid phospho-ERK rebound upon KRAS G12C inhibition.^23^ Additionally, KRAS G12C-mutant colorectal tumors demonstrate distinct co-mutation patterns, with significantly higher rates of STK11 (20.6%) and KEAP1 (15.4%) alterations compared to non-G12C KRAS-mutant tumors, both of which are independently associated with poor prognosis and resistance to targeted therapies.^24^ Large-scale genomic analyses reveal that 46.5-53.8% of KRAS G12C colorectal cancers harbor putative resistance alterations at baseline, which may limit therapeutic efficacy and contribute to the observed worse overall survival.^25^

This meta-analysis establishes KRAS G12C mutation status as a prognostic biomarker that should inform patient counseling strategies and setting up expectations. The 28% increased mortality risk provides clinicians with valuable prognostic information that can guide discussions about treatment urgency, importance of clinical trial enrollment, and the timing of palliative care consultations. Furthermore, it provides a foundation for risk-stratified approaches that recognize KRAS G12C-mutant mCRC as a distinct molecular subset requiring expedited access to KRAS-targeted therapies and more intensive monitoring strategies.^6^

Another potential clinical implication becomes particularly relevant in the context of current treatment paradigms of mCRC patients with *KRAS G12C*. KRAS G12C inhibitors are currently approved in patients with mCRC who have received prior treatment with fluoropyrimidine-, oxaliplatin-, and irinotecan-based chemotherapy.^4^^,^^5^^,^^26^ The worse overall survival associated with KRAS G12C mutation indirectly suggests inferior outcomes to currently available standard of care treatments. It is also well recognized that a substantial subset of patients with mCRC may not have the opportunity to receive second- or third-line therapies.^27^ These observations have prompted consideration of whether KRAS G12C inhibitors should be integrated earlier in the treatment sequence.

However, several important caveats must be acknowledged. First, the combination of KRAS G12C inhibitors with anti-EGFR antibodies, while representing the current approved approach, has demonstrated modest efficacy in colorectal cancer. The CodeBreaK 300 trial demonstrated improved PFS with sotorasib plus panitumumab versus standard therapy (5.6 vs 2.0 months), yet the clinical benefit remains modest.^4^ Concerns about censoring methodology have been raised, and the OS analysis—though deemed underpowered—did not reach significance.^4^^,^^28^ Similarly, the KRYSTAL-1 trial demonstrated response rates of 34% for adagrasib plus cetuximab, which, while meaningful, underscores the ongoing challenge of treating KRAS-mutant colorectal cancer.^29^ Second, historical experience with combining small molecule targeted therapies with cytotoxic chemotherapy in colorectal cancer has been largely unsuccessful with few important exceptions (including Breakwater and CRDF-004 trials),^4^^,^^6^ suggesting that simply moving KRAS G12C inhibitors to earlier lines in combination with chemotherapy may not improve outcomes and provide meaningful benefit.

Furthermore, the potential risks of earlier integration of KRAS G12C inhibitors warrant careful consideration. Moving a targeted therapy with a distinct toxicity profile to the frontline setting could compromise patients’ ability to complete standard cytotoxic chemotherapy, which remains the backbone of mCRC treatment. The toxicities associated with KRAS G12C inhibitors, including gastrointestinal effects (diarrhea, nausea, vomiting), hepatotoxicity, and skin toxicities (primarily from the anti-EGFR component), may be additive or synergistic with chemotherapy-related adverse events.^4^^,^^30^ Additionally, there is currently no prospective evidence demonstrating that earlier use of KRAS G12C inhibitors improves overall survival compared to their use in later lines; this remains a theoretical extrapolation from prognostic data.

Important limitations of our analysis must be acknowledged. First, all included studies were retrospective in design, introducing potential selection bias and confounding. Second, the studies were conducted during the pre-KRAS G12C inhibitor era, and the observed survival disadvantage reflects outcomes with standard chemotherapy regimens. Extrapolating these findings to predict differential benefit from KRAS G12C inhibitors or other novel therapies should be done with caution, as the prognostic significance of a mutation under standard treatment may not directly translate to predictive significance for targeted therapy response. Third, heterogeneity in treatment regimens, lines of therapy, and patient populations across studies may limit generalizability. Fourth, while moderate heterogeneity was observed (*I*^2^ = 54.3%), sensitivity analyses consistently demonstrated the robustness of our findings.

KRAS G12C mutation status, as a prognostic biomarker, does have implications for clinical trial design, selection, and enrollment decisions. Patients with KRAS G12C mutations represent a high-risk population who may be appropriate candidates for clinical trials investigating novel approaches, including KRAS G12C inhibitor combinations in earlier treatment settings.^5^^,^^31^ However, the decision to enroll patients in such trials should be made in the context of clinical equipoise, recognizing that the benefit of these approaches remains unproven.

## Conclusion

This meta-analysis provides strong evidence that *KRAS G12C* mutations confer worse prognosis compared to non-G12C RAS mutations in mCRC, with a 28% increased risk of mortality. This establishes KRAS G12C as an independent adverse prognostic biomarker in mCRC. These findings have important implications for patient counseling and risk stratification. However, the translation of prognostic significance into therapeutic decision-making requires caution. While the poor prognosis of KRAS G12C-mutant mCRC may provide rationale for prioritizing clinical trial enrollment and considering earlier access to KRAS G12C inhibitors, the potential benefits must be weighed against the risks of moving these therapies to earlier lines including toxicity concerns, potential compromise of standard chemotherapy delivery, and the absence of prospective data demonstrating survival benefit from earlier integration. Prospective randomized trials are needed to determine whether earlier use of KRAS G12C inhibitors improves outcomes in this high-risk population.

## Supplementary Material

oyag219_Supplementary_Data

## Data Availability

The data supporting this systematic review and meta-analysis are derived from published studies and are available within the article and its supplementary materials.
