# Supplementary material for: Prognostic significance of KRAS G12C versus non-G12C RAS mutations in metastatic colorectal cancer: a systematic review and meta-analysis
Source: Oncologist. 2026 Jun 2;31(7):oyag219. doi: 10.1093/oncolo/oyag219 (PMC13283479; doi:10.1093/oncolo/oyag219)
Supplement: oyag219_Supplementary_Data [file oyag219_supplementary_data.docx]

**Supplementary Materials**

Supplemental Table S1. Detailed Itemization of the Newcastle-Ottawa Scale (NOS) Risk of Bias Assessment

| Study | Representativeness of Exposed Cohort | Selection of Non-Exposed Cohort | Ascertainment of Exposure | Outcome Not Present at Start | Comparability of Cohorts | Assessment of Outcome | Adequate Follow-Up Duration | Adequacy of Follow-Up | Total Score (Max 9 pts) |
| --- | --- | --- | --- | --- | --- | --- | --- | --- | --- |
| Chida 2021 | 1 | 1 | 1 | 1 | 2 | 1 | 1 | 1 | 9 |
| Giampieri 2021 | 1 | 1 | 1 | 1 | 2 | 1 | 1 | 1 | 9 |
| Fakih 2022 | 1 | 1 | 1 | 1 | 2 | 1 | 1 | 1 | 9 |
| Osterlund 2022 | 1 | 1 | 1 | 1 | 2 | 1 | 1 | 1 | 9 |
| Schirripa 2020 | 1 | 1 | 1 | 1 | 2 | 1 | 0 | 1 | 8 |
| Henry 2021 | 1 | 1 | 1 | 1 | 1 | 1 | 1 | 1 | 8 |
| Ottaiano 2020 | 1 | 1 | 1 | 1 | 2 | 1 | 1 | 1 | 9 |
| Li 2022 | 1 | 1 | 1 | 1 | 1 | 1 | 1 | 1 | 8 |
| Koulouridi 2022 | 1 | 1 | 1 | 1 | 0 | 1 | 1 | 0 | 6 |
| Lavacchi 2022 | 1 | 1 | 1 | 1 | 0 | 1 | 1 | 1 | 7 |
| Khamis 2025 | 1 | 1 | 1 | 1 | 0 | 1 | 1 | 0 | 6 |
| Uson Jr 2018 | 0 | 1 | 1 | 1 | 2 | 0 | 1 | 1 | 7 |
| Peeters 2012 | 1 | 1 | 1 | 1 | 0 | 1 | 1 | 1 | 7 |
| Contreras Toledo 2024 | 1 | 1 | 1 | 1 | 2 | 1 | 1 | 1 | 9 |

Scores for each item are 0 or 1, except for 'Comparability of Cohorts' which can be awarded up to 2 points.

Supplemental Table S2. Individual Study Median Overall Survival and Progression-Free Survival

| **Study** | **KRAS G12C (n)** | **Non-G12C RAS (n)** | **G12C mOS (months)** | **Non-G12C mOS (months)** | **G12C mPFS (months)** | **Non-G12C mPFS (months)** |
| --- | --- | --- | --- | --- | --- | --- |
| Chida 2021 | 45 | 651 | 21.1 | 27.3 | 9.4 | 10.8 |
| Giampieri 2021 | 15 | 105 | 37.3 | 24.7 | 8.6 | 9.8 |
| Fakih 2022 | 238 | 2947 | 16.1 | 18.3 | 7.4 | 9.0 |
| Osterlund 2022 | 103 | 881 | 26.2 | 22.0 | 12.7 | 11.7 |
| Schirripa 2020 | 145 | 694 | 28.9 | 36.7 | NR | NR |
| Henry 2021 | 187 | 720 | 21.2 | 31.6 | 6.4 | NR |
| Ottaiano 2020 | 13 | 144 | 5.0 | 17.3 | NR | NR |
| Li 2022 | 30 | 613 | 27.0 | 29.0 | 11.0 | NR |
| Koulouridi 2022 | 28 | 539 | NR | NR | NR | NR |
| Lavacchi 2022 | 13 | 169 | 52.9 | 20.6 | NR | NR |
| Khamis 2025 | 24 | 297 | 65.0 | 52.0 | NR | NR |
| Uson Jr 2018 | 17 | 72 | NR | NR | NR | NR |
| Peeters 2012 | 12 | 114 | NR | NR | NR | NR |
| Contreras Toledo 2024 | 33 | 459 | 10.6 | NR | 4.8 | 6.0 |

mOS: median overall survival; mPFS: median progression-free survival; NR: Not Reported

Supplemental Figure S1. Newcastle-Ottawa Scale Quality Assessment Summary


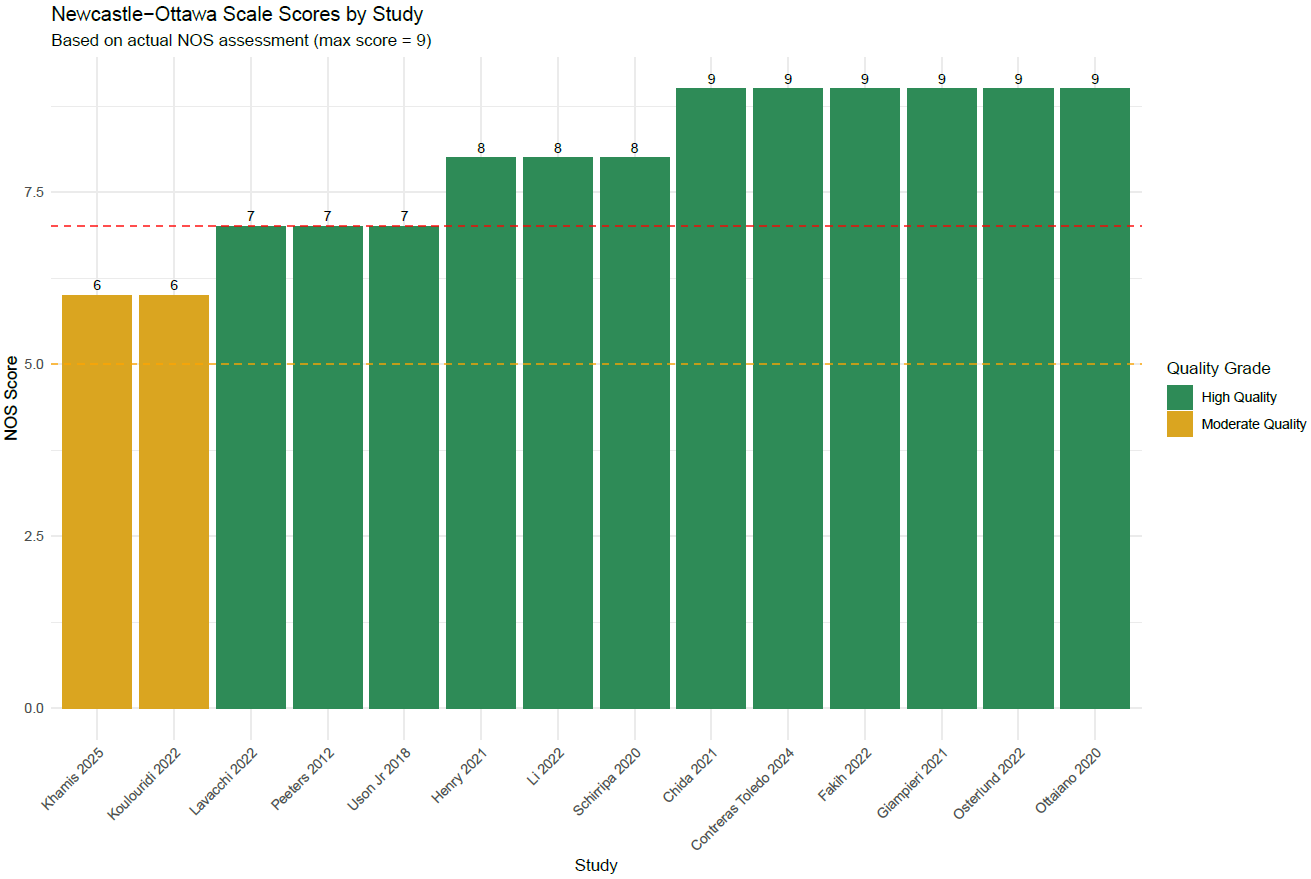


Supplemental Figure S2. Forest Plot for High-Quality Studies


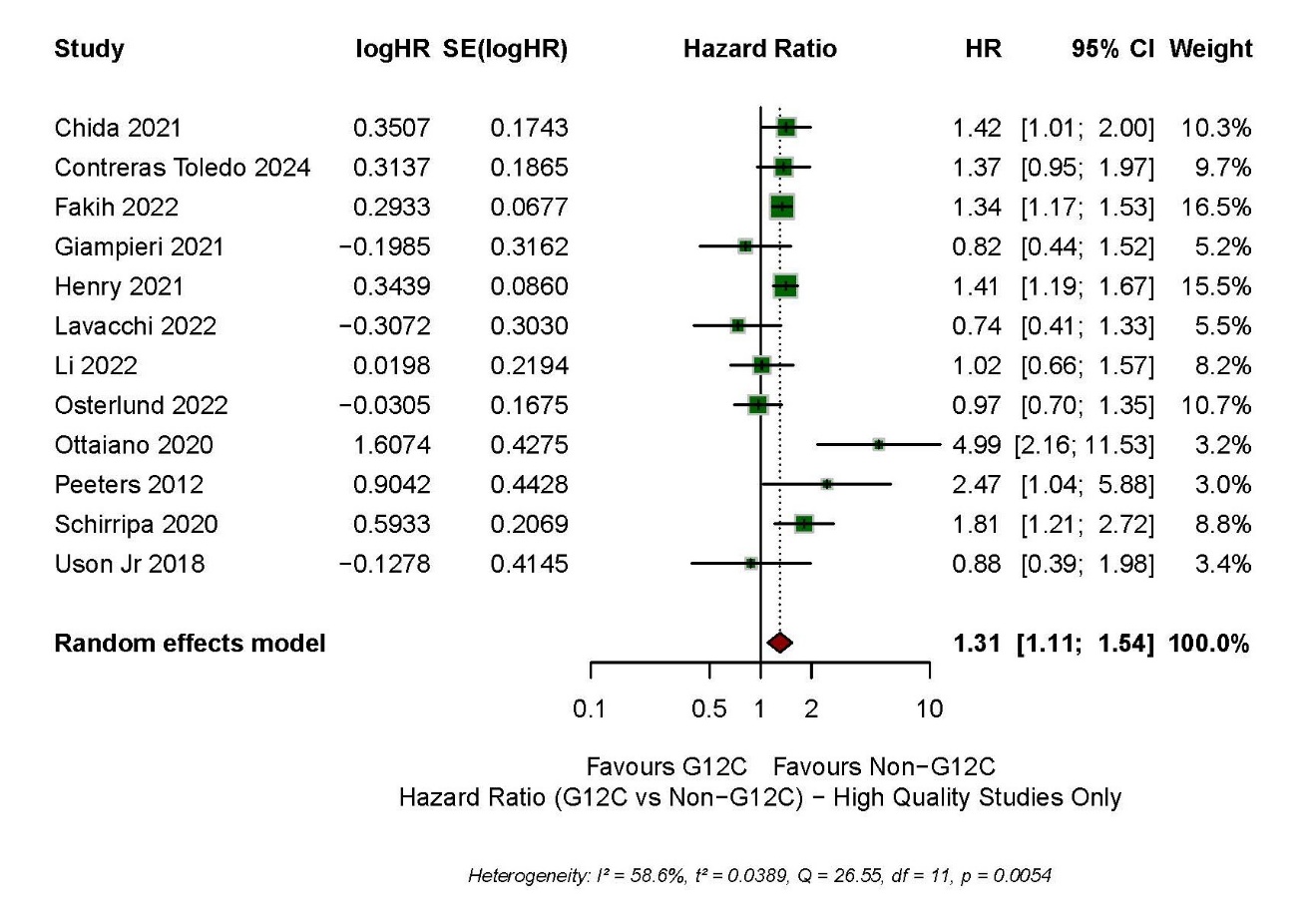


Forest Plot of Hazard Ratios for Overall Survival, Restricted to High-Quality Studies (NOS ≥ 7).

Supplemental Figure S3. Funnel Plot for Publication Bias Assessment


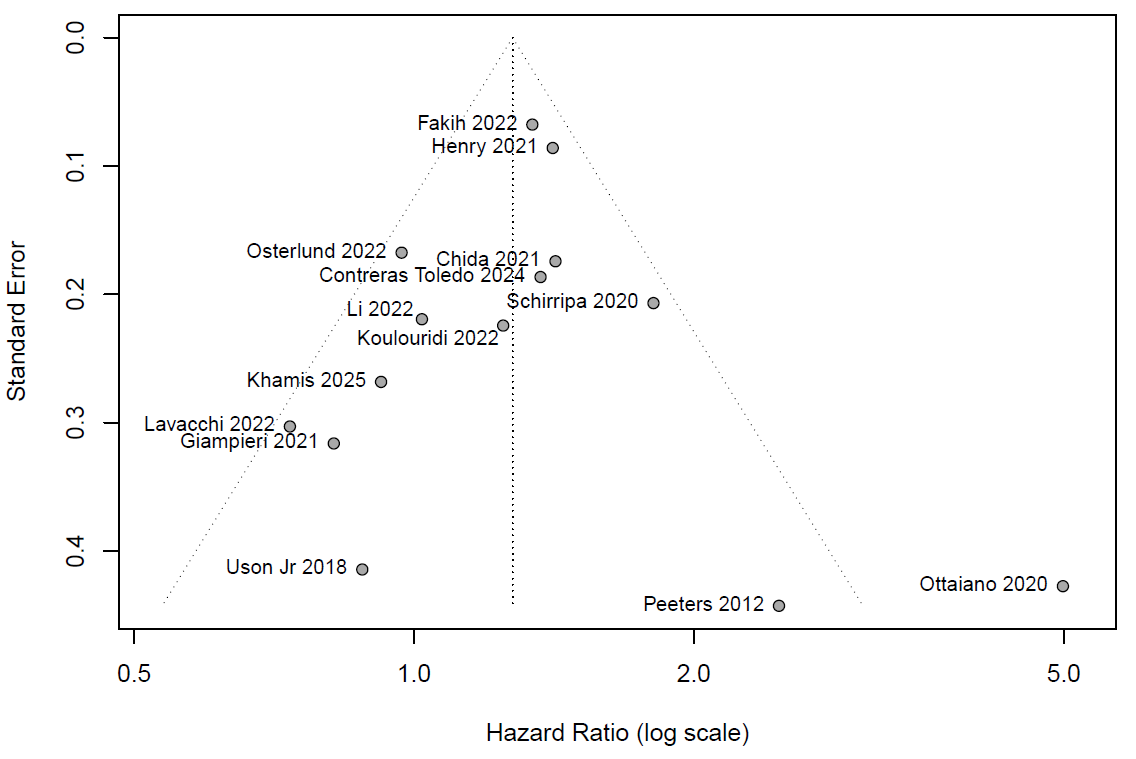


Funnel Plot of Standard Error by Log Hazard Ratio for Overall Survival.

Supplemental Methods:

PubMed Search Strategy

(

  ("Colorectal Neoplasms"[Mesh] OR "colorectal cancer"[tiab] OR "colon cancer"[tiab] OR "rectal cancer"[tiab] OR "colorectal adenocarcinoma"[tiab] OR "CRC"[tiab])

  AND

  ("Neoplasm Metastasis"[Mesh] OR "metastatic"[tiab] OR "advanced"[tiab] OR "unresectable"[tiab] OR "stage IV"[tiab] OR "stage 4"[tiab] OR "mCRC"[tiab])

  AND

  (

    ("Proto-Oncogene Proteins p21(ras)"[Mesh] OR "Proto-Oncogene Proteins B-raf"[Mesh])

    OR

    ("KRAS"[tiab] OR "NRAS"[tiab] OR "BRAF"[tiab] OR "RAS"[tiab])

    OR

    ("G12C"[tiab] OR "non-G12C"[tiab] OR "non G12C"[tiab] OR "G12V"[tiab] OR "G12D"[tiab] OR "V600E"[tiab])

    OR

    ("mutat*"[tiab])

  )

  AND

  (

    ("Survival Analysis"[Mesh] OR "Prognosis"[Mesh] OR "Mortality"[Mesh] OR "Proportional Hazards Models"[Mesh])

    OR

    ("overall survival"[tiab] OR "OS"[tiab] OR "hazard ratio"[tiab] OR "HR"[tiab] OR "survival"[tiab] OR "prognostic"[tiab] OR "prognosis"[tiab] OR "outcome"[tiab])

  )

)

NOT

(

  "review"[Publication Type] OR "editorial"[Publication Type] OR "comment"[Publication Type] OR "letter"[Publication Type]

)

Google Scholar Search Strategy

("metastatic colorectal cancer" OR "advanced colorectal cancer" OR "stage IV colorectal cancer" OR mCRC)

AND ("KRAS G12C" OR "KRAS p.G12C")

AND ("overall survival" OR "OS" OR "prognosis" OR "hazard ratio" OR "Kaplan-Meier" OR "median survival")

AND ("non-G12C" OR "other KRAS" OR NRAS OR "RAS mutation")
